# What happens after HIV self-testing? Results from a longitudinal cohort of Chinese men who have sex with men

**DOI:** 10.1186/s12879-019-4455-8

**Published:** 2019-09-14

**Authors:** Weiming Tang, Wenting Huang, Haidong Lu, Bolin Cao, Dan Wu, Jason Ong, Hongyun Fu, Ye Zhang, Bin Yang, Cheng Wang, Wei Ma, Chongyi Wei, Joseph D. Tucker

**Affiliations:** 1 Dermatology Hospital, Southern Medical University Guangzhou, Guangzhou, China; 2 University of North Carolina at Chapel Hill Project-China, Guangzhou, 510095 China; 3 SESH Study Group, Guangzhou, China; 4 School of Public Health, University of North Carolina at Chapel Hill, Chapel Hill, USA; 5 College of Mass Communication, Shenzhen University, Shenzhen, China; 6 Division of Community Health and Research, Eastern Virginia Medical School, Norfolk, Virginia, USA; 7 School of Public Health, Southern Medical University, Guangzhou, China; 8 School of Public Health, Shandong University, Jinan, China; 9 Rutgers University, Brunswick, NJ USA; 10 Faculty of Infectious and Tropical Diseases, London School of Hygiene and Tropical Medicine, London, UK

**Keywords:** Condom use, Facility-based testing, HIV self-testing (HIVST), Longitudinal study, Men who have sex with men

## Abstract

**Background:**

HIV self-testing (HIVST) is a promising approach to expand HIV testing. HIVST is a process in which a person performs an HIV test and interprets the result. Negative HIVST results may encourage men who have sex with men (MSM) to use HIV prevention services. The objective of this study was to examine behaviors (e.g., facility-based HIV testing, condom use) after a negative HIVST test result among Chinese MSM.

**Methods:**

We collected data from MSM in eight Chinese cities over a 12-month period. This is a secondary analysis of longitudinal cohort data collected as part of an intervention trial to increase HIV testing. Men completed a survey that described sociodemographic information, sexual behaviors, HIV self-testing, and facility-based HIV testing. Men who completed at least one follow-up survey were included in this analysis. Generalized linear mixed models were used to evaluate whether HIVST increased subsequent facility-based HIV testing and consistent condom use.

**Results:**

We included 1219 men. Most men (78.7%) were under 30 years old and had never been married (87.0%). 755 (61.9%) men tested for HIV and 593 (49.3%) men self-tested during the study period. At baseline, among men who had never been tested for HIV, 44.9% (314/699) initiated HIVST during the study period. HIVST was associated with subsequent facility-based testing (aOR of 1.87, 95% CI: 1.47–2.37). HIVST was also associated with subsequent consistent condom use (aOR = 1.53, 95% CI: 1.13–2.06).

**Conclusion:**

HIVST was associated with subsequent facility-based HIV testing and consistent condom use. HIVST may enhance uptake of related HIV prevention services at facilities, suggesting the need for more implementation research.

## Background

HIV testing is the first step in the HIV care continuum and an important component of comprehensive HIV services [1]. However, approximately one-quarter of people living with HIV (PLWH) do not know their serological status [2]. This lack of awareness contributes to ongoing HIV transmission in many key populations [3, 4]. In China, men who have sex with men (MSM) have a high burden of HIV infection [5, 6]. For example, HIV prevalence reached 8% among MSM in 2015 [6] and a high HIV incidence has been observed [7]. However, a systematic review of 54 studies examining MSM HIV testing in China showed only 47% of MSM had ever tested for HIV [8]. Low rates of HIV testing among MSM in China are likely related to limited testing options, incomplete protection of privacy, and HIV test-related stigma [9–11].

HIV self-testing (HIVST) is a promising approach for expanding HIV testing. HIVST refers to a process in which a person collects his or her own specimen (oral fluid or blood), performs an HIV test, and interprets the result [12, 13]. HIVST can be undertaken alone or with a friend, partner, family member, or other person [14, 15]. HIVST enables individuals to test themselves at their convenience. The World Health Organization recommends HIVST as an additional approach for HIV testing [14]. Studies have suggested that HIVST is acceptable and feasible across a wide range of populations and places [16–18]. HIVST provides an additional opportunity for identifying people living with HIV who have not yet been diagnosed [14]. For example, half of the newly identified MSM HIV cases in 2017–2018 in Zhuhai, a Chinese city, were identified through HIVST [19]. HIVST likely increases HIV testing rates, including first-time HIV testing [20]. One study conducted by our study team found that 60% of MSM HIV self-testers reported that HIV self-testing was their first ever HIV test [21].

However, many MSM who receive HIV testing do not change sexual behaviors or access other HIV prevention services. Few studies have examined what happens to facility-based HIV test uptake or consistent condom use after HIVST. This issue is particularly important among people who receive a negative HIVST and may use their negative HIVST result to justify persistent high-risk behaviors. Better understanding what happens after a negative HIVST result can help us to improve HIV prevention services and linkage to clinical services. The objective of this study was to examine behaviors (e.g., facility-based HIV testing, consistent condom use) after a negative HIVST test result among a longitudinal cohort of Chinese MSM.

## Methods

This is a secondary analysis of cohort data collected as part of a stepped-wedge randomized controlled trial that evaluated the effectiveness of a crowdsourced intervention to promote HIV testing among Chinese MSM [22, 23]. Crowdsourcing has a group attempt to solve a problem or a component of a problem, then share solutions [24]. HIVST promotion materials were developed through a series of crowdsourcing contests. The trial collected data from MSM over a 12-month period starting on July 2016 in eight Chinese cities (Guangzhou, Jiangmen, Shenzhen and Zhuhai in Guangdong Province, Jinan, Jining, Qingdao and Yantai in Shandong Province) (Clinical Trials ID: NCT02796963). These cities were chosen based on the following criteria: 1) previous CDC MSM sentinel surveillance site; 2) capacity for campaign implementation; 3) capacity for intervention implementation at the community level.

### Participant recruitment and follow-up

Detailed information about recruitment and follow-up has been described elsewhere [23]. In brief, a banner link for recruitment was sent to registered Blued, a large gay social network app, users living in selected cities [25]. MSM who were interested in the survey clicked the survey link and were directed to the baseline survey that was hosted by Sojump (Shanghai, China). To be eligible, the men needed to be 16 years old or older, live in one of the eight cities with no plan to move to another city in the next 12 months, be HIV negative or not aware of their HIV status, not have HIV testing in the last 3 months, had anal sex with a man at least once during their lifetime, and be willing to provide their cell phone number for follow-up. Men were assigned into four groups based on the city from which they were recruited. Men were exposed to the HIV testing intervention depending on their group assignment. Cities were randomized into respective intervention groups independently. Eligible men signed an online informed consent and completed the baseline survey instrument. The crowdsourced intervention included an online HIV testing campaign, an online HIVST testing service with free home delivery, and local participatory activities. Data collection occurred at baseline and then quarterly, for a total of five times over a 12-month period. Men diagnosed with HIV infection were censored. Men received 50 RMB (~ USD 8.50) for enrolling in the study and participating in each follow-up. The incentives were distributed to men through WeChat, an encrypted instant messaging service. Since the four groups received the same intervention during follow-up, we combined men from the four groups for this analysis. The analysis was restricted to men with at least one follow-up observation.

### Measures

The baseline survey collected socio-demographic information, sexual behaviors, HIV/STI testing history, self-reported HIV status, and whether or not HIV test results were disclosed to their most recent partner. The socio-demographic information included age (in years), city of residence, residency status (resident in the local city, resident of other cities in the same province, or resident of other province), marital status (never married, currently married, divorced or widowed), educational level (high school or less, some college, completed college or more than college), and annual income ($2500 USD or below, $2501–8500 USD, $8501–14,000 USD, or more than $14,000 USD). We also collected information regarding self-identified sexual orientation (gay, bisexual, or unsure), ever disclosed sexual orientation to anyone except male partners (dichotomous), and ever disclosed sexual orientation to healthcare providers (dichotomous).

We asked about several sexual behaviors and HIV testing behaviors. We asked men if they had sex with any male partner in the last 3 months (dichotomous) and whether they consistently used a condom with a male partner in the last 3 months (dichotomous). At each follow-up period, we asked about consistent condom use with male partners in the past 3 months. In addition, men were asked if they had ever tested for HIV (dichotomous). Among those who had received HIV testing, we asked about ever tested at facility-based sites (dichotomous) and ever self-testing (dichotomous). At each follow-up period, we asked about HIV facility-based testing in the last 3 months and HIVST in the last 3 months.

We adapted validated scales to measure anticipated HIV stigma [26], HIV testing social norms (attitudes toward HIV testing using six items with a 4-point Likert scale) [27], HIV testing self-efficacy [28], and community engagement in sexual health during the follow-up period [29]. Detailed information on these measures has been reported elsewhere [22, 23, 30].

### Statistical analysis

We used descriptive analysis to examine the distribution of socio-demographic characteristics and sexual behaviors of men who completed at least one follow-up survey. Generalized linear mixed models were used to evaluate the association of HIVST (as a dependent variable) with HIV testing within 3 months following HIVST, facility-based testing within 3 months following HIVST, and condom use with male partners within 3 months following HIVST, after adjusting for secular trend (random effect), age, marital status, and income. Our study is a secondary analysis of a stepped wedge randomized controlled trial. Our findings were similar between the two provinces and we did not adjust for province in our data analysis. All data analyses were completed using SAS 9.4 (SAS, Cary, NC, USA).

### Ethics statement

Ethical approval was obtained from ethics review committees at the Dermatology Hospital of Southern Medical University (Guangzhou, China), the University of North Carolina at Chapel Hill (Chapel Hill, North Carolina), the University of California, San Francisco (San Francisco, California), and Rutgers University (Piscataway, New Jersey) prior to the survey launch. Inform consent was obtained from all the men by e-signing the online informed consent.

## Results

Overall, 1381 MSM who has not tested for HIV in the last 3 months were recruited. Among this group of men, 1219 finished at least one follow-up survey and were included in the data analysis (Table 1).

**Table 1:** Baseline demographic characteristics of men who completed at least one follow-up survey in China, 2016–2017 (*N* = 1219)

Variables	n	%	
Age Group (years)	< 20	197	16.2
	20–29	762	62.5
	30–39	207	17.0
	40 or above	53	4.4
Residence status	The sampling city	376	30.8
	Other cities in the province	463	38.0
	Other provinces	380	31.2
Marital Status	Never married	1061	87.0
	Currently married	110	9.0
	Divorced or widowed	48	3.9
Educational level attained	High school or below	429	35.2
	Some College	348	28.6
	College or above	442	36.3
Annual income (USD)	2500 or below	263	21.6
	2501-8500	654	53.7
	8501-14,000	192	15.8
	> 14,000	110	9.0
Province	Guangdong	603	49.5
	Shandong	616	50.5
Sexual Orientation	Gay	862	70.7
	Bisexual	304	24.9
	Unsure	53	4.4
Ever disclosed sexual orientation	Yes	794	65.1
	No	425	34.9
Ever disclosed sexual orientation to health providers^a^	Yes	251	31.6
	No	543	68.4
Had a male partner in the last 3 months	Yes	657	53.9
	No	562	46.1
Consistently used a condom with male partners in the last 3 months^b^	Yes	333	50.7
	No	324	49.3
Ever tested for HIV	Yes	520	42.7
	No	699	57.3
Ever self-tested for HIV	Yes	202	16.6
	No	1017	83.4
HIVST as first HIV testing before the study^c^	Yes	114	21.9
	No	406	78.1

Note:

^a^Among people reported ever disclosed sexual orientation to anyone else except male partner

^b^Among people who had a male partner in the last 3 months

^c^among people who tested for HIV before (520)

### Social-demographic characteristics and behaviors

At baseline, about half of the men were recruited from Guangdong Province (49.5%) while the other half were recruited from Shandong Province (50.5%). The majority of the 1219 men were under 30 years old (78.7%), residents of the province (68.8%), never married (87.0%), and attended some college (64.8%). In addition, only 9.0% of the men had an annual income greater than USD 14,000.

Most (70.7%) men self-identified as gay, and 65.1% reported that they disclosed their sexual orientation to anyone other than their male sexual partners. Among men who disclosed their sexual orientation (*n* = 794), 31.6% disclosed their sexual orientation to health care providers.

Approximately half of men (53.9%) reported that they had sex with male partners in the last 3 months. Approximately half of men (50.7%) consistently used a condom when having sex with male partners. At baseline, 53.9% (657/1219) of men reported that they engaged in anal sex with male partners in the last 3 months. This proportion was 58.1% (651/1120), 56.7% (617/1088), 58.7% (613/1044) and 60.6% (625/1031) during the first, second, third and fourth follow-up period, respectively (Additional file 1).

#### HIV testing

Seven hundred and fifty-five (61.9%) men tested for HIV and 593 (49.3%) men self-tested during the study period. Of the 1219 people who completed at least one follow-up survey, about 13.0% (158/1219), 36.8% (449/1219), 49.7% (606/1219) and 61.9% (755/1219) of men tested once (including facility-based testing and self-testing) during the first 3 months, first 6 months, first 9 months and in 12 months of follow-up, respectively.

**Fig. 1:**
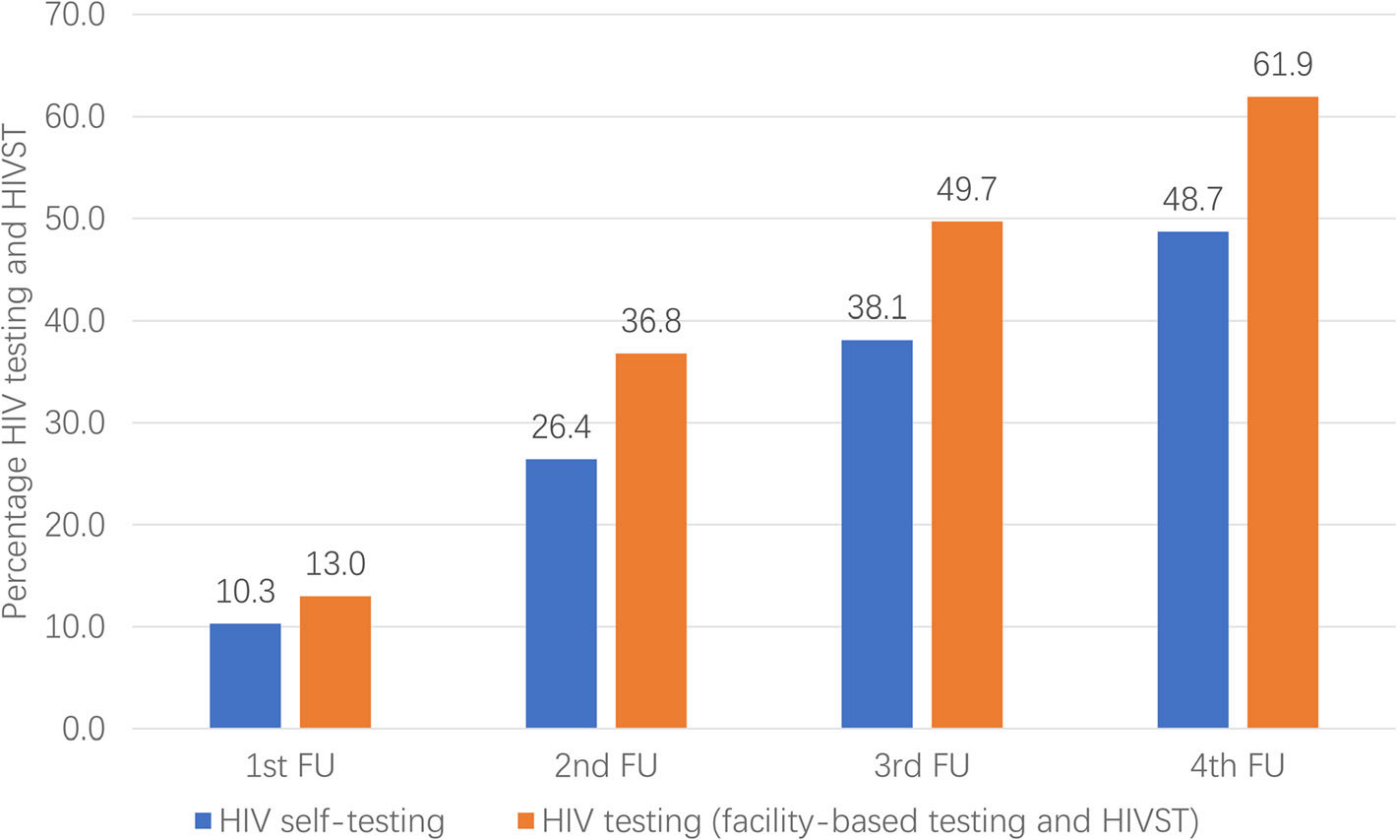
Cumulative HIV testing (including HIV self-testing) uptake among Chinese MSM, 2016–2017 (*N* = 1219)

In addition, about 10.3% (126/1219), 26.4% (322/1219), 38.1% (464/1219), and 48.7% (593/1219) of men had self-tested at least once during the first 3 months, first 6 months, first 9 months and in 12 months of follow-up, respectively (Fig. 1). Among the 699 men who never tested for HIV before, 314 (44.9%) used HIVST during the study period. Among men who used HIVST, 89 (15.0%) men reported that their HIV testing results were positive. Of these 89 men, 43 (48.3%) reported that they confirmed their testing results at facility-based sites within the same three-month follow-up period. All of them were confirmed to be living with HIV infection.

#### Facility-based testing and condom use following HIVST

The generalized linear mixed model showed that a negative HIVST result was associated with subsequent overall HIV testing and subsequent facility-based testing, with adjusted ORs (aORs) of 2.45 (95% CI: 1.99, 3.01) and 1.46 (95% CI: 1.15, 1.86), respectively. We also found that HIVST was associated with subsequent consistent condom use with male partners, with an OR of 1.53 (95% CI: 1.13, 2.06) (Table 2).

**Table 2:** The association of HIVST on following overall HIV testing, facility-based testing, and condom use among MSM in China, 2016–2017. (*N* = 1219)

Variables		Measures (95% CIs)	
		Crude OR (95% CIs)^a^	Adjusted OR (95% CIs)^b^
Overall subsequent HIV testing	No	Ref	
	Yes	2.50 (2.04, 3.06)	2.45 (1.99, 3.01)
Subsequent facility-based testing	No	Ref	
	Yes	1.87 (1.47, 2.37)	1.46 (1.15, 1.86) ^c^
Subsequent HIV self-testing	No	Ref	
	Yes	1.38 (1.04,1.82)	1.42 (1.07,1.88)
Consistent condom use with a male partner	No	Ref	
	Yes	1.56 (1.16, 2.10)	1.53 (1.13, 2.06)
Community engagement	Not Increased	Ref	
	Increased	1.13 (0.32, 3.99)	1.21 (0.31, 4.66)
		Crude scale difference (95% CIs) for continuous outcomes	Adjusted scale difference (95% CIs)^b^
Anticipated Stigma		−0.010 (−0.055, 0.035)	−0.008 (−0.053, 0.037)
Social norm		−0.019 (−0.008, 0.065)	−0.019 (− 0.009, 0.064)
Self-efficacy		− 0.022 (− 0.019, 0.062)	− 0.021 (− 0.019, 0.062)

Note:

^a^Models were adjusted by time

^b^Models were adjusted by time, age (continuous), marital status, income, education level and intervention in the trial, as compared to the value measured in the previous follow-up

^c^Previous facility-based testing was also adjusted

## Discussion

HIVST can reach hard-to-find key populations who have never used facility-based HIV testing [14, 31]. Previous studies focused on evaluating the feasibility and acceptability of HIVST [32], assessing the testing accuracy of HIVST kits [33], and piloting HIVST distribution models [34]. This study adds to the literature by evaluating facility-based testing and condom use following a negative HIVST result. Our findings suggest that HIVST is associated with subsequent facility-based HIV testing and consistent condom use. Our data have implications for expanding HIV prevention services among MSM in China.

We found that men with a negative HIVST result were more likely to receive subsequent facility-based HIV testing. This finding contrasts an Australian MSM study which reported that facility-based HIV test uptake did not change following HIV self-testing [35]. This difference may be due to variations in HIVST models [36] or the already high HIV facility-based HIV testing rates among MSM in Australia [35]. Our finding is similar to other research which suggest that HIVST can serve as an entry point for pre-exposure prophylaxis [37] and male circumcision [38]. Other facility-based HIV prevention services such as STI testing could also be integrated with HIVST.

We found that men who received HIVST were subsequently more likely to report consistent condom use. A previous review found mixed evidence regarding whether HIVST is associated with subsequent condom use [39]. However, studies suggest that an additional HIV prevention benefit of HIVST may be the option to test at the point of sex [32, 40]. Testing at the point of sex refers to individuals using HIVST to screen potential sex partners immediately before sex and then making a decision whether or not to have sex based on the test results [18]. This finding underscores the potential behavioral advantages of HIVST.

This study has policy and research implications. From a policy perspective, HIVST is a potential tool for promoting other HIV prevention services, especially among MSM who do not currently access facility-based services. The potential for HIVST to drive facility-based testing may help to reach MSM, identify HIV cases, link people to care, and provide PrEP services. National-level policies supporting HIVST in the broader context of HIV prevention services may help to formalize this integration. From a research perspective, the public health benefits of HIVST suggest the need for more implementation research [21, 41]. Optimizing the scale-up of smaller, community-based HIVST pilots into larger programs will require pragmatic trials and process evaluations [32, 42].

This study has several limitations. First, men in this study were recruited through a single geosocial networking mobile phone application, Blued. Our sample of MSM were young and well-educated. This group of MSM may be more likely to use HIVST compared to other groups of MSM in China. Making inferences based on our data to other MSM groups should be done with caution. Second, the information collected in this study mainly relied on self-report, leading to social desirability bias and information bias. However, data from the stepped wedge trial indicated that there was a high agreement between self-reported data and data from photo-verification. Third, some people included in the analysis did not attend all of the follow-up surveys. As a result, our study may underestimate HIVST uptake rates. Last, the HIV self-testing rate reported in this study was collected in a trial setting, which may over-estimate uptake. However, we did not aim to compare this rate with other studies, and this finding suggests strong demand for HIVST among Chinese MSM.

## Conclusions

HIVST is a useful tool for expanding HIV testing. Our data show that HIVST is associated with subsequent facility-based testing and consistent condom use among MSM in China. HIVST could serve as an entry point for other HIV prevention services and contribute to comprehensive HIV services.

## Supplementary information

**Additional file 1. **Proportion of people who have had sex with male partners in the last 3 months among Chinese MSM, 2016–2017 (*N* = 1219).

## Data Availability

The datasets used and analyzed during the current study are available from the corresponding author on reasonable request.

## Abbreviations

HIV: Human Immunodeficiency virus
HIVST: HIV self-testing
MSM: Men who have sex with men
OR: Odds ratio
PLWH: People living with HIV
RMB: Ren Min Bi
USD: US dollars
