# Supplementary figures and images for: What happens after HIV self-testing? Results from a longitudinal cohort of Chinese men who have sex with men
Source: BMC Infect Dis. 2019 Sep 14;19:807. doi: 10.1186/s12879-019-4455-8 (PMC6744670; doi:10.1186/s12879-019-4455-8)

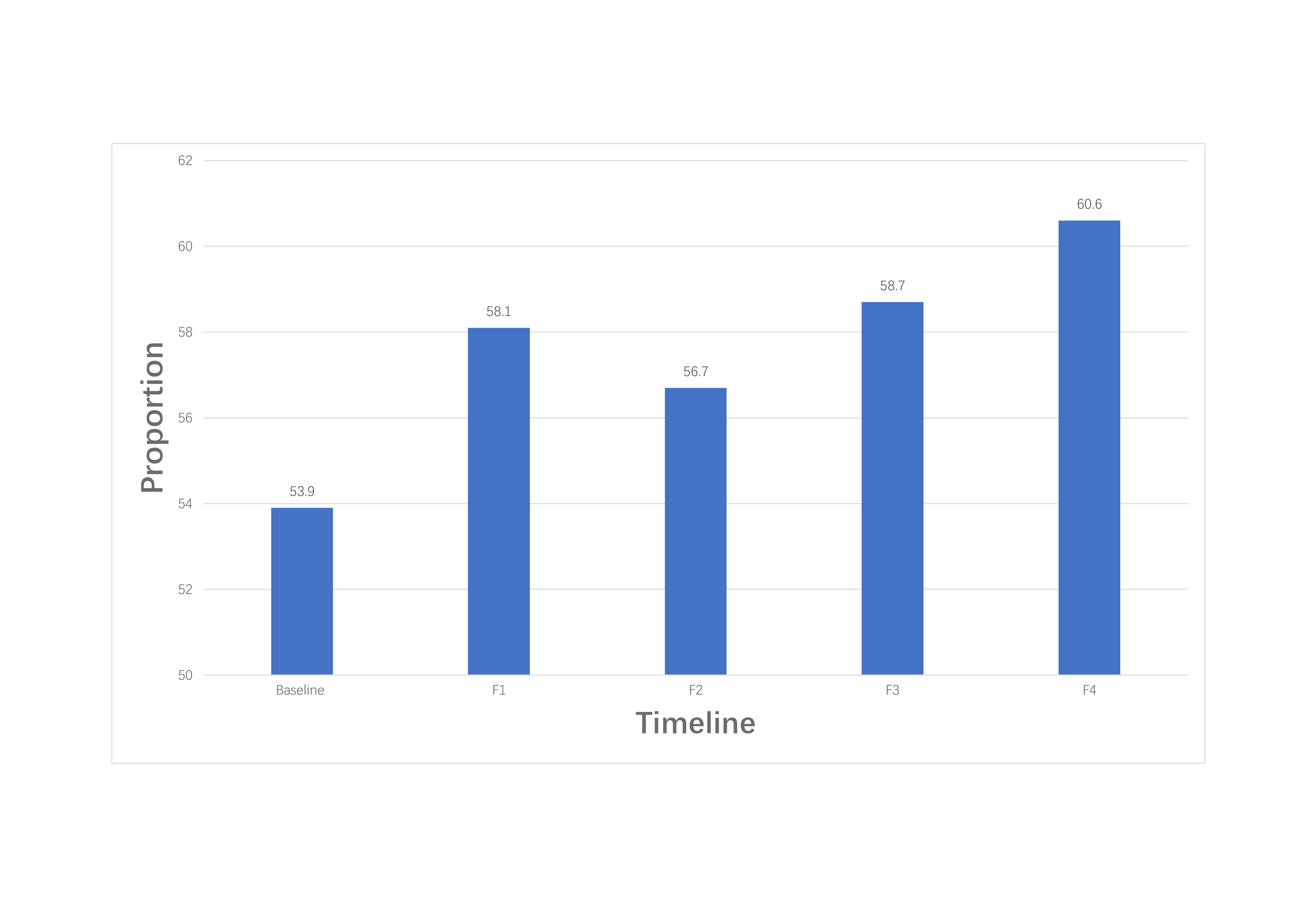

Supplement: Supplementary file 1 — Additional file 1. Proportion of people who have had sex with male partners in the last 3 months among Chinese MSM, 2016–2017 (N = 1219). [file 12879_2019_4455_MOESM1_ESM.tiff]
